# Incremental Activation Detection for Real-Time fMRI Series Using Robust Kalman Filter

**DOI:** 10.1155/2014/759805

**Published:** 2014-01-06

**Authors:** Liang Li, Bin Yan, Li Tong, Linyuan Wang, Jianxin Li

**Affiliations:** China National Digital Switching System Engineering and Technological Research Center, Zheng Zhou 450002, China

## Abstract

Real-time functional magnetic resonance imaging (rt-fMRI) is a technique that enables us to observe human brain activations in real time. However, some unexpected noises that emerged in fMRI data collecting, such as acute swallowing, head moving and human manipulations, will cause much confusion and unrobustness for the activation analysis. In this paper, a new activation detection method for rt-fMRI data is proposed based on robust Kalman filter. The idea is to add a variation to the extended kalman filter to handle the additional sparse measurement noise and a sparse noise term to the measurement update step. Hence, the robust Kalman filter is designed to improve the robustness for the outliers and can be computed separately for each voxel. The algorithm can compute activation maps on each scan within a repetition time, which meets the requirement for real-time analysis. Experimental results show that this new algorithm can bring out high performance in robustness and in real-time activation detection.

## 1. Introduction

Functional magnetic resonance imaging (fMRI) offers a noninvasive method in studying human brain functions by recording blood-oxygen-level-dependent (BOLD) signal changes related to neuronal activity across the brain with high spatial resolution [1]. Real-time fMRI (rt-fMRI) is a method to assess the acquired data for evidence of an experimentally induced effect at every intracerebra voxel individually and simultaneously. In rt-fMRI, data are processed as fast as they are acquired [2]. For real-time fMRI applications, mapping the activations within a repetition time makes it possible to interact with fMRI experiments in a much more efficient way [3]. Online functional mapping enables researchers to monitor data quality, evolve experimental protocols more rapidly, perform interactive experimental paradigms for neurological investigation [4], achieve neurofeedback by providing feedback of brain activation to the subject in real time [5], which may have potential use in clinical applications [6].

In common fMRI experiments, MRI scanner acquires whole brain data at an interval of 2 seconds, also called repetition time. To meet the real-time requirements, all the processing steps of real-time fMRI need to be completed within a repetition time. Simple real-time fMRI processing steps consist of data reconstruction, spatial realignment (head motion correction), and statistical analysis. Among them, incremental statistical analysis on each voxel of the fMRI dataset will result in huge computational costs. To overcome the computational costs of the statistical analysis, a number of incremental activation detection algorithms have been developed for rt-fMRI applications.

Cox et al. [7] proposed the first real-time incremental activation detection algorithm and the correlation based activation detection methods are not able to model multiple experimental and confounding effects simultaneously. Gembris et al. [8] proposed correlation analysis method using sliding window technique. Friston et al. [9] proposed the general linear model (GLM), which can be used as a unified framework in the analysis of fMRI data and support multiple experimental design, but it cannot be used to rt-fMRI applications, because it needs all of the data to do the statistical analysis. The widely used correlation based techniques are special cases of GLM with a white noise model for the temporal errors in the signal. Bagarinao et al. [10] proposed an algorithm using an orthogonalization procedure to estimate the coefficient of general linear models. Roche et al. [11] proposed an algorithm using extended kalman filter (EKF) method to fit a general linear model on fMRI time courses. This technique adopts the GLM-AR model under the assumption that the fMRI noise is significantly autocorrelated. The extended kalman filter is able to fit incrementally GLM coefficients along with the one-order autoregressive noise model. In this paper, we mainly focus on the EKF method, because it requires low computation costs and memory costs, and the design matrix can be assembled incrementally, which makes it possible for more complex interactive experiment design.

The fMRI time series has low signal to noise ratio [12]. The noise in the fMRI signal is complicated. It includes not only the usual MRI sensor noise but also physiological fluctuations that affect the signal. The fluctuations of MRI scanner or severe head motion may generate sparse noise in the signal. In our model, under the assumption that the sparse noise will not change the noise distribution, a variation is added to the extended kalman filter to handle the additional measurement noise term, that is, sparse; this term can be used to model the sparse measurement outliers.

In recent years, with the developments of convex optimization, Mattingley and Boyd [13] created a robust kalman filter by replacing the standard measurement update, which can be interpreted as the result of solving a similar convex minimization problem, which includes an *l*_1_ term to handle the sparse noise. We use the robust kalman filter method to solve the value of the sparse term in our model.

## 2. Method

Real-time fMRI signal is three-dimensional volume data at each scan during a repetition time and the intensity of each voxel represents the blood oxygen level associated with neural activities. Data of each incoming fMRI scans is spatially aligned with the first scan of the series. Voxel values at different scan time points are arranged to time sequence, forming the measured time series. Each voxel will form a time series, and the length of time series is growing with the time increasing. The time series of each voxel can be calculated independently, so in the following discussion we only consider the situation of a single voxel time series.

### 2.1. Extended Kalman Filter Incremental Detection

Roche et al. [11] proposed an extended kalman filter based algorithm to fit incrementally the general linear model along with a one-order autoregressive noise model.

The general linear model (GLM) explains the measured time series *y* = [*y*_1_, *y*_2_,…, *y*_*n*_]^*T*^ in terms of a linear combination of the explanatory variables *X*_*p*_ = [*x*_1*p*_, *x*_2*p*_,…, *x*_*np*_]^*T*^ plus an error term. The explanatory variable contains paradigm-related regressors based on the experiment design and signal characteristics and regressors are obtained by convolving the different stimulation onsets with a canonical hemodynamic response function. Model the low-frequency drift, hence enabling us to “detrend” the signal (we use polynomials up to order three). Then combine the explanatory variables into a design matrix *X* = [*X*_1_, *X*_2_,…, *X*_*p*_]. The GLM can be expressed as

(1) $$\begin{array}{c} y=X\beta +\varepsilon . \end{array}$$

The design matrix is *X* = [*X*_1_, *X*_2_,…, *X*_*p*_], where *X*_*p*_ is the explanatory variable, *β* = [*b*_1_, *b*_2_,…, *b*_*p*_]^*T*^ contains (unknown) parameters which represent the coefficients of the explanatory variables. The design matrix contains paradigm-related regressors based on the experiment design. The regressors can be obtained by convolving the different stimulation onsets with a canonical hemodynamic response function, or modeling the low-frequency drift to detrend the signal.

In the GLM-AR model, it is assumed that *ε* is a stationary Gaussian zero mean AR(1) random process and the relationship between *ε*_*i*_ and *ε*_*i*−1_ can be expressed as.

(2) $$\begin{array}{c} \varepsilon_{i}=a\varepsilon_{i-1}+n_{i}. \end{array}$$

*a*  is an (unknown) autocorrelation parameter, and *n*_*i*_ is a white noise with instantaneous Gaussian distribution *N*(0, *σ* ^2^).

Alexis Roche proved that the maximum likelihood estimate of (*β*, *a*) can be computed independently from *σ* and they found that one can reach the maximum of the likelihood function by finding the minimum of *ρ*_*i*_(*β*, *a*):

(3) $$\begin{array}{c} \rho_{i}\left(\beta ,a\right)=\{\begin{array}{cc} \sqrt{1-a^{2}}\left(y_{1}-x_{1}^{T}\beta \right), & \text{if}\;\;i=1\\ y_{i}-x_{i}^{T}\beta -a\left(y_{i-1}-x_{i-1}^{T}\beta \right), & \text{otherwise}, \end{array} \end{array}$$

where *x*_*i*_ = [*x*_*i*1_, *x*_*i*2_,…, *x*_*ip*_]^*T*^ denote the values of explanatory variables at time *i*.

First, they combine the parameters to be estimated *β* and *a* into a (*p* + 1) × 1 state vector *b* = [*β*;*a*]^*T*^ and assume that, at time *i*, the current estimate of *b* is ${\hat{b}}_{i-1}=[{\hat{\beta }}_{i-1};{\hat{a}}_{i-1}]$.

Secondly, linearize the error function *ρ*_*i*_(*β*, *a*) to *ρ*_*i*_(*b*) around the current estimate using a first-order Taylor expansion:

(4) minρi(b)=qi−uiTb,

where $q_{i}=y_{i}-{\hat{a}}_{i-1}x_{i-1}^{T}\beta_{i-1}$ (*i* = 1, *q*_1_ = *y*_1_), $u_{i}={\left[x_{i}-{\hat{a}}_{i-1}x_{i-1},y_{i-1}-x_{i-1}^{T}{\hat{\beta }}_{i-1}\right]}^{T}$ (*i* = 1, *u*_*i*_ = [*x*_1_,0]^*T*^).

Finally, they solved the (nonlinear) least-squares regression problem by means of an EKF. The EKF updates the parameters using the following recursion:

(5) $$\begin{array}{c} {\hat{b}}_{i}={\hat{b}}_{i-1}+\left(q_{i}-u_{i}^{T}{\hat{b}}_{i-1}\right)k_{i},\\ k_{i}={\left(1+u_{i}^{T}\Sigma_{i-1}u_{i}\right)}^{-1}\Sigma_{i-1}u_{i},\\ \Sigma_{i}=\left(I_{p+1}-k_{i}u_{i}^{T}\right)\Sigma_{i-1}, \end{array}$$

where *k*_*i*_ is the kalman gain in *i*th step and Σ_*i*_ denote the normalized posterior covariance matrix of *b* given the information available at time *i*.

At time *i*, the posterior covariance matrix of the state vector is approximated by $\text{Cov}\left(b_{i}\right)\approx {\hat{\sigma }}_{i}^{2}\Sigma_{i}$ and the incremental update rule for *σ* is as follows:

(6) $$\begin{array}{c} {\hat{\sigma }}_{i}^{2}=\frac{i-1}{i}\left[{\hat{\sigma }}_{i-1}^{2}+{\left(q_{i}-u_{i}^{T}{\hat{b}}_{i-1}\right)}^{2}\left(1-u_{i}^{T}k_{i}\right)\right]. \end{array}$$

The number of explanatory variables is *p*, and *β* is the *p* × 1 vector which contains the coefficients related to the explanatory variables. Σ_*i*_^*p*×*p*^ is obtained by extracting the left superior *p* × *p* block from the matrix ${\hat{\sigma }}_{i}^{2}\Sigma_{i}$. For a given contrast vector *c*, we can identify the voxels that show a contrasted effect:

(7) $$\begin{array}{c} T_{i}={\left(c^{T}\Sigma_{i}^{p\times p}c\right)}^{-1/2}c^{T}\beta \\ P_{i}\left(c^{T}\beta >0\right)\approx \frac{1}{2}\left[1+\mathrm{erf}\left(\frac{t_{i}}{\sqrt{2}}\right)\right], \end{array}$$

where *t*_*i*_ = (*c* ^*T*^Σ_*i*_^*p*×*p*^ *c*)^−1/2^ *c* ^*T*^ *β* and *erf*(·) is the error function. Testing for positive activations can be achieved at any time *i* by thresholding the image of *t*_*i*_-statistics.

### 2.2. Outlier Detection Method

The sensor failures or measurement outliers will cause the sparse measurement noise and they may cause rapid degrade on the detection performance. We derive a new algorithm to detect the outliers in order to eliminate the effect on the kalman filter algorithm.

We suppose that there is a sparse term *z*_*i*_ which is always zero, and it has no effect on the distribution of the total noise; then the general linear model can be modified as

(8) yi=xiβ+εi+ziρi(β,a,zi)={1−a2(y1−x1Tβ−z1)if    i=1yi−xiTβ−zi−a(yi−1−xi−1Tβ−zi−1)otherwise.

Then linearize the *ρ*_*i*_(*β*, *a*, *z*_*i*_) to *ρ*_*i*_(*b*, *z*_*i*_):

(9) $$\begin{array}{c} \rho \left(b,z_{i}\right)\approx q_{i}-u_{i}^{T}b-z_{i}, \end{array}$$

where *q*_*i*_ = *y*_*i*_ − *a*_*i*−1_ *x*_*i*−1_^*T*^ *β*_*i*−1_ and *u*_*i*_ = [*x*_*i*_−*a*_*i*−1_ *x*_*i*−1_^*T*^,*y*_*i*−1_−*x*_*i*−1_^*T*^ *β*_*i*−1_−*z*_*i*−1_]^*T*^, *z*_*i*_ is an unknown variable at time *i*, while *z*_*i*−1_ is an known variable at time *i*.

The linearized constraint equation is approximate as follows:

(10) $$\begin{array}{c} q_{i}=u_{i}^{T}b_{i}+z_{i}+v_{i}. \end{array}$$

The measurement update step of standard kalman filter algorithm is essentially an optimization problem, and the linearized parameter optimization problem can be described as follow:

(11) minviTV−1vi+(b−b^i−1)TΣ−1(b−b^i−1)subject  to qi=uiTb+vi,

where *b* and *v*_*i*_ are the unknown parameters to be estimated, Σ denotes the steady-state error covariance associated with predicting the next state *b*, and the measurement is *q*_*i*_ and the measurement noise term *v*_*i*_ is a white noise with instantaneous Gaussian distribution *N*(0, *V*).

We use the robust kalman filter method to detect the outlier hidden in the measurement by replacing the measurement update with the solution of a similar convex minimization problem, which includes an *l*_1_ term to handle the sparse noise.

To (approximately) handle the additional sparse noise term *z*_*i*_, we modify the kalman filter measurement update step. The modified optimization problem is as follows:

(12) minviTV−1vi+(b−b^i−1)TΣ−1(b−b^i−1)+λ||zi||1subject  to qi=uiTb+vi+zi.

In the optimization problem (12), *b*, *z*_*i*_, and *v*_*i*_ are the variables to be estimated.

To solve this convex optimization problem, we adopt a fast transform method proposed by Mattingely and Boyd [13], which makes problem solving become more effective:

(13) $$\begin{array}{c} L_{i}=\Sigma_{i}u_{i}{\left(u_{i}^{T}\Sigma_{i}u_{i}+V\right)}^{-1}\\ Q_{i}={\left(I-u_{i}^{T}L_{i}\right)}^{T}V^{-1}\left(I-u_{i}^{T}L_{i}\right)+L_{i}^{T}\Sigma^{-1}L_{i}\\ e_{i}=q_{i}-u_{i}^{T}b_{i-1}. \end{array}$$

After the transformation, the original problem was transformed into an equivalent convex quadratic program problem:

(14) $$\begin{array}{c} {\left(e_{i}-z_{i}\right)}^{T}Q_{i}\left(e_{i}-z_{i}\right)+\lambda {||z_{i}||}_{1}. \end{array}$$

With variable *z*_*i*_, solving the convex quadratic program, we can achieve the sparse noise *z*_*i*_ at each time. Fortunately, for one voxel at time *i*, the size of *e*_*i*_, *z*_*i*_ and *Q*_*i*_ is 1 × 1. Hence this optimization problem is equivalent to solve the solution of a piecewise-quadratic function. This problem has an analytical solution, means we can use analytical expression instead of the searching optimization loop.

### 2.3. Robust Extended Kalman Filter

The standard kalman filter consists of alternating time and measurement updates. Since *β* is slowly varying parameter, so it remains unchanged in the time update step. Both of the time and measurement updates are derived by the minimum mean-square error estimates of *b*. As is shown in (5), the measurement update equation is as follows:

(15) $$\begin{array}{c} {\hat{b}}_{i}={\hat{b}}_{i-1}+\left(q_{i}-u_{i}^{T}{\hat{b}}_{i-1}\right)k_{i}. \end{array}$$

After solving the problem, we achieve an estimate outlier value ${\hat{z}}_{i}$ and then combine it into the update step:

(16) $$\begin{array}{c} {\hat{b}}_{i}={\hat{b}}_{i-1}+\left(q_{i}-u_{i}^{T}{\hat{b}}_{i-1}-{\hat{z}}_{i}\right)k_{i}. \end{array}$$

Furthermore, we combine the ${\hat{z}}_{i}$ into the incremental update rule for *σ*:

(17) $$\begin{array}{c} {\hat{\sigma }}_{i}^{2}=\frac{i-1}{i}\left[{\hat{\sigma }}_{i-1}^{2}+{\left(q_{i}-u_{i}^{T}{\hat{b}}_{i-1}-{\hat{z}}_{i}\right)}^{2}\left(1-u_{i}^{T}k_{i}\right)\right]. \end{array}$$

For the real-time *t*-test, the method is exactly the same as Alexis Roche's method.

Finally, the algorithm recursion is summarized as follows:

(18) qi=yi−a^i−1xiTβi−1 (i=1,z1=y1)ui=[xi−a^i−1xi−1,yi−1−xi−1Tβ^i−1−zi−1]T(i=1,ui=[x1,0]T)Li=Σi−1ui(uiTΣi−1ui+V)−1Qi=(I−uiTLi)TV−1(I−uiTLi)+LiTΣi−1−1Liei=qi−uiTbi−1minzi (ei−zi)TQi(ei−zi)+λ||zi||1b^i=b^i−1+(qi−uiTb^i−1−z^i)kiki=(1+uiTΣi−1ui)−1Σi−1uiΣi=(Ip+1−kiuiT)Σi−1.

## 3. Result

The algorithm is tested on a single run from an fMRI experiment involving a visual and auditory task. The protocol is block design, and the run consisted of 10 blocks, each block including one activation epoch (20 s) and one control epoch (10 s). We aim at finding the voxels associated with the visual and auditory function; each activation epoch, the visual stimulus, and auditory stimuli are present, but the intensity is different. The data has an abrupt head motion during the scan in the 45th repetition time (TR) and the head motion caused severe motion artifacts. The repetition time was 2 seconds for a total of 152 scans. Functional images have 80∗80∗33 voxels.

**Figure 1:**
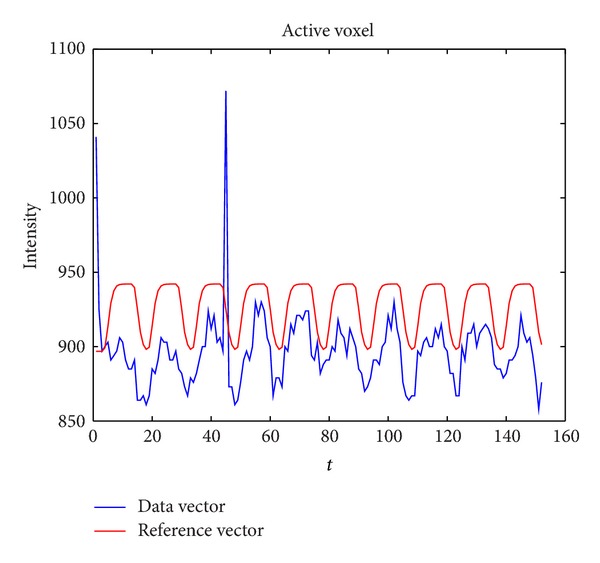


As is shown in Figure 1, the red curve is the reference vector obtained by convolving the stimulation onset with a canonical hemodynamic response function, which is assumed the time series should be; the blue curve is the time series data of an active voxel; obviously there is an outlier at the 45th TR.

**Figure 2:**
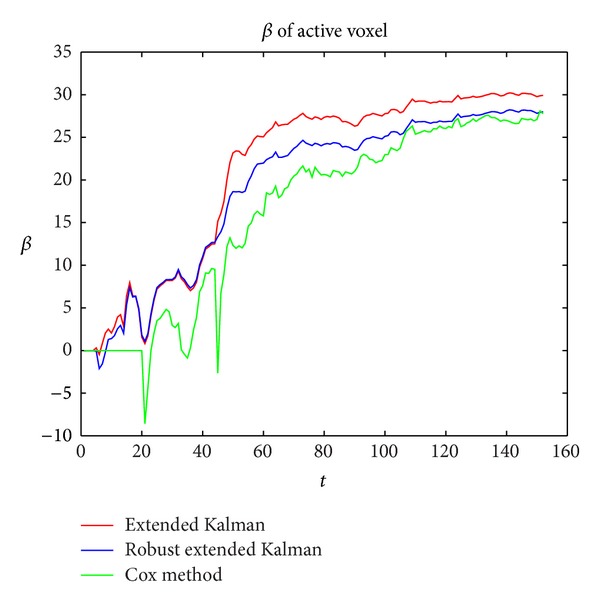


In Figure 2, all of the three curves tend to be stabilized and the correlation coefficient *β* of activate voxel is all converge to around 26. The green curve presents Cox method, and it has a great drop at the outlier scan, and the extended kalman filter method has an abnormal rise at the 45th TR, while our method has no great fluctuation at the outlier scan. This shows its robustness to the sparse noise.

**Figure 3:**
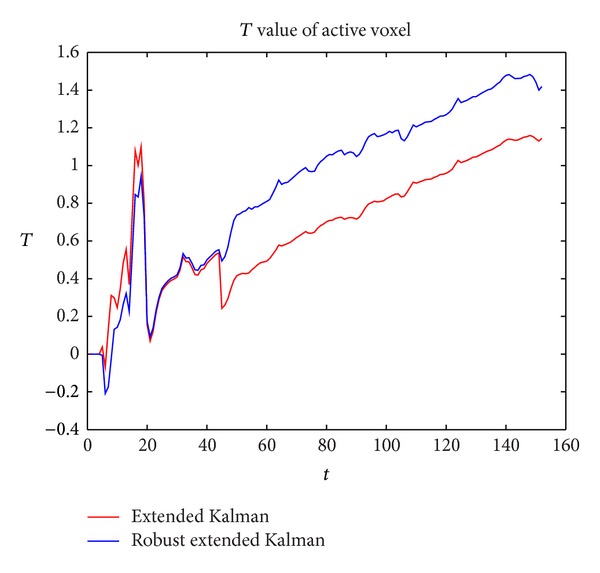


Cox method derived a threshold for active detection, so there is no *T* value in their method.  Figure 3 shows the comparison of the *t*-test value between the Robust extended kalman filter and the extended kalman filter. We can see that both of the two algorithms have significant decrease on the outlier. Obviously, our method is more stable at the outlier scan and has a higher *T* value after the outlier scan.

**Figure 4:**
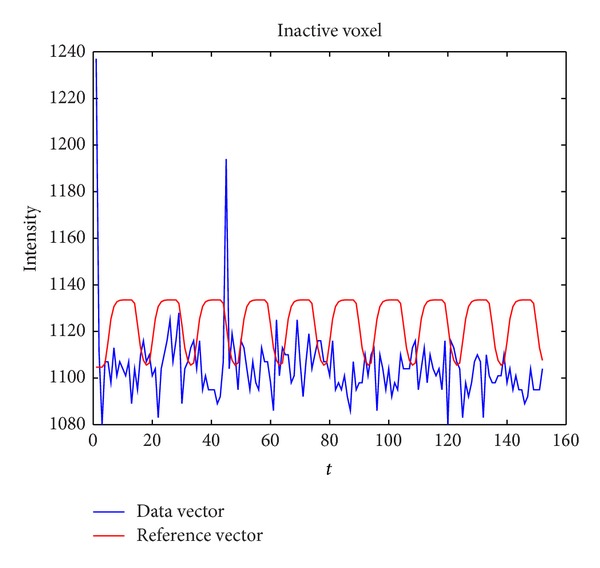


We also tested the algorithm on an inactivate voxel. In Figure 4, the blue curve is the time series of an inactive voxel and at the 45th TR there is also an outlier.

**Figure 5:**
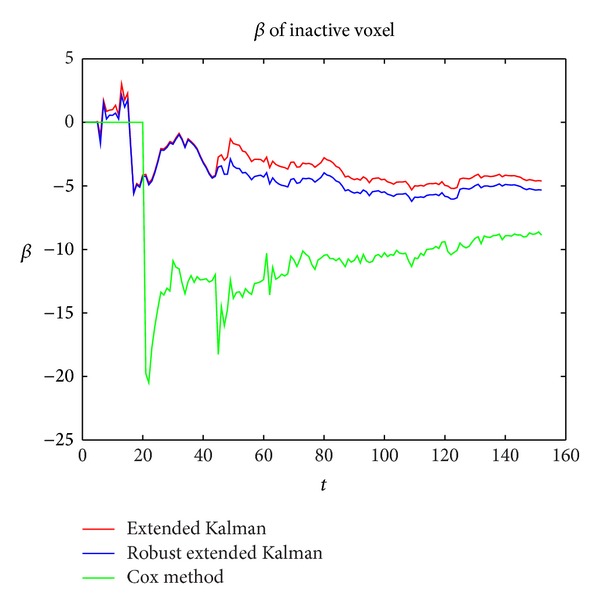


The *t*-test value of the inactive voxel should be low, as is shown in Figure 5; the correlation curves of the three methods tend to be stable below zero. There is a big fluctuation at the outlier scan according to Cox method and our method finally obtained a stable value lower than the extended kalman method. Before the outlier scan, the correlations of the two methods show little difference. After the outlier scan, our method becomes lower than the extended kalman method.

**Figure 6:**
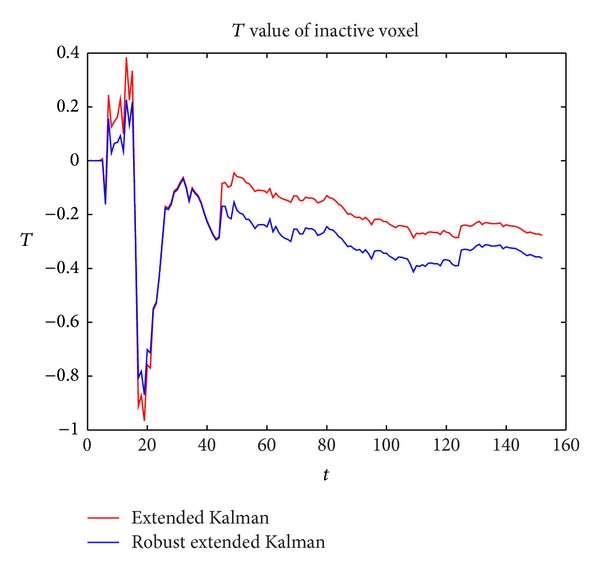


In Figure 6, a comparison of the *T* value between our method and the extended kalman filter method is shown. We can see that *T* value of both the two algorithms has fluctuations at the outlier, and the fluctuations of our method are smaller than those of extended kalman filter method. Our method tends to be stable at a lower level. This again shows our new method's robustness to the sparse noise in the inactive voxel.

**Figure 7:**
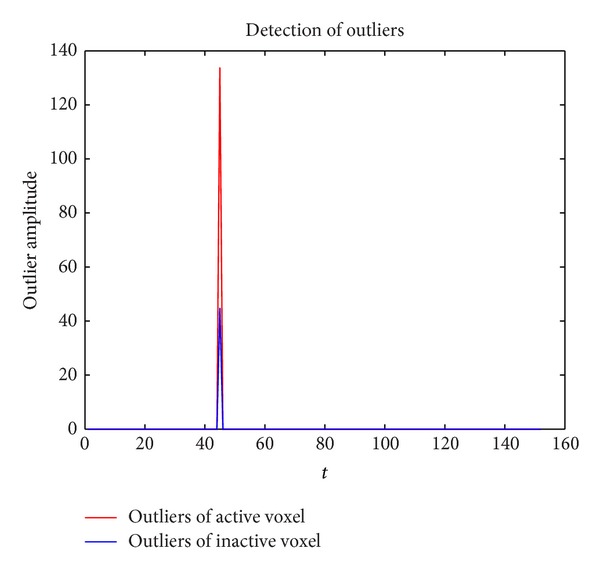


Figure 7 is the result of outlier detection, as is shown in Figures 1 and 4; the original signal contains a big outlier at the 45th TR and the margin of the two outlier is different. Our outlier detection algorithm detect two outliers from both the active and inactive voxel at 45th TR, it is shown to be robust for both the active and inactive voxels.

**Figure 8:**
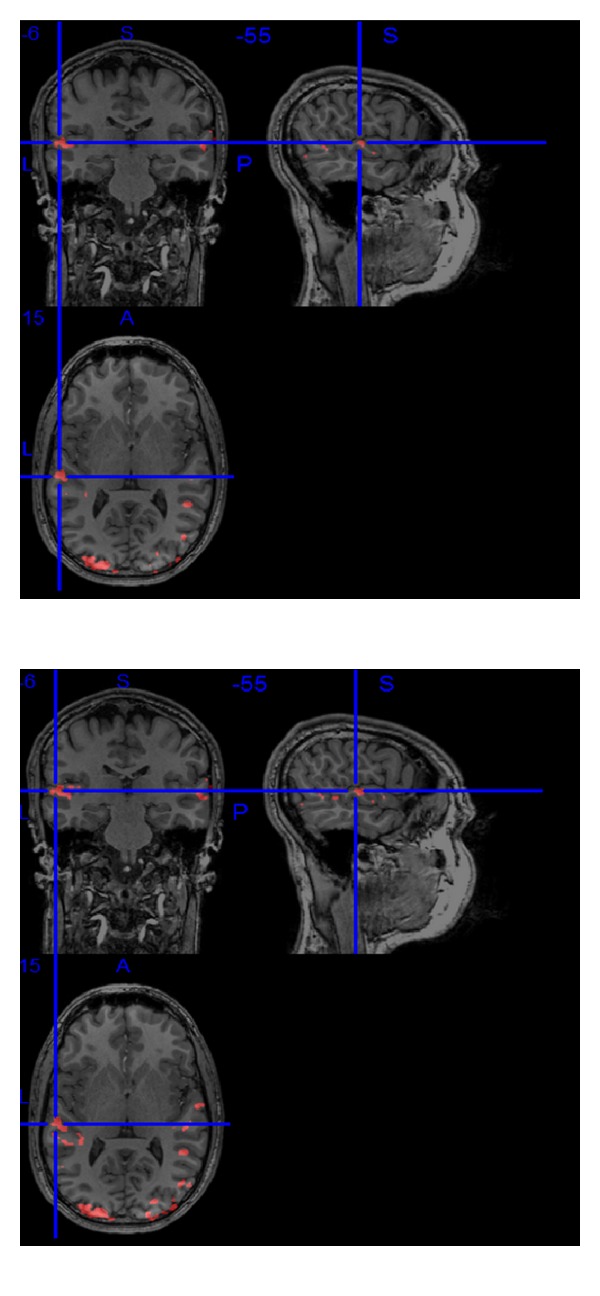


We have applied our detection algorithm to the whole brain data, in the case where threshold value the same, the final activation maps are shown in Figure 8; left half is the activation result achieved by the extended kalman algorithm and right half is the result of the algorithm described in this paper. As expected for a visual auditory task, activations are found in the visual cortex and auditory cortex on both sides. This means that, under the same threshold of *T* value, we can get more voxels associated with the task.

We have a C++ version of the algorithm, and we test it on a 16-core processor workstation, in which computing hole brain (80∗80∗32) voxels costs 0.3 s to 0.4 s; there is plenty of time to do some other processes. Our algorithm can be used on the real-time fMRI application.

## 4. Discussion

In our algorithm, parameter *λ* needs to be defined before the experiment. In the optimization object function, the parameter *λ* can be regarded as the weight of the sparse term. With a large *λ*, the outlier detection algorithm will find nothing and result zero in *z*_*i*_, the measurement update will be the same as the extended kalman filter method. So if no outlier is detected, the result of the algorithm will be the same as that of in the extended kalman filter. Small *λ* will make the algorithm modify the measurement update frequently. Under this situation, the mistaken outlier will have an effect on the noise distribution and cause the unstability of the kalman filter. Here we can regard *λ* as the detection threshold and by holding a large threshold we can detect the obvious outlier, which may be caused by the machine failure. The value of *λ* in our algorithm is in a wide range. We suggest taking a relatively large *λ* to test if the result is stable. In this experiment, we test *λ* from 50 to 300 and the algorithm can detect the outlier and the result is robust to the outlier.

The fMRI signal has a low signal to noise ratio, so we cannot give an exact threshold value and this algorithm provides a method to threshold the signal, and we can even achieve the value of the outlier and use it to modify the detection algorithm. fMRI experiment is complex, which needs cooperation with subject and operator; any problem of them may cause the experiment to fail. Our algorithm can detect this kind of failure and it can detect the outliers, which may have a great effect on follow processes, where we can stop the experiment to check the problem or just mark it and eliminate the outlier data after the experiment.

In future works, we will explore a proper method to determine *λ*, just enough to detect the outlier but not cause the unstability of the kalman filter algorithm.

## 5. Conclusion

The robust kalman filter is introduced in the activation detection of fMRI experiment. Convex optimization method is used to modify the extended kalman filter by introducing a sparse noise term. The robustness of our method to the sparse noise is improved. Moreover, the performance of the proposed method does not degrade rapidly when disturbances are involved. When applied to the time series voxels, our method can obtain more stable *t*-test value in both activate and inactivate voxels. The proposed algorithm can also detect outliers, which may have a great effect on following processes. The detected outlier information can be used to reject or modify the bad data, which may have potential benefit for real-time applications that require higher data quality.

## References

[1] Logothetis NK (2003). MR imaging in the non-human primate: studies of function and of dynamic connectivity. *Current Opinion in Neurobiology*.

[2] Weiskopf N, Sitaram R, Josephs O (2007). Real-time functional magnetic resonance imaging: methods and applications. *Magnetic Resonance Imaging*.

[3] Christopher deCharms R (2008). Applications of real-time fMRI. *Nature Reviews Neuroscience*.

[4] LaConte SM (2011). Decoding fMRI brain states in real-time. *NeuroImage*.

[5] Weiskopf N (2011). Real-time fMRI and its application to neurofeedback. *NeuroImage*.

[6] Birbaumer N, Murguialday AR, Weber C, Montoya P (2009). Chapter 8 neurofeedback and brain-computer interface: clinical applications. *International Review of Neurobiology*.

[7] Cox RW, Jesmanowicz A, Hyde JS (1995). Real-time functional magnetic resonance imaging. *Magnetic Resonance in Medicine*.

[8] Gembris D, Taylor JG, Schor S, Frings W, Suter D, Posse S (2000). Functional magnetic resonance imaging in real time (FIRE): sliding-window correlation analysis and reference-vector optimization. *Magnetic Resonance in Medicine*.

[9] Friston KJ, Holmes AP, Worsley KJ, Poline J-P, Frith CD, Frackowiak RSJ (1994). Statistical parametric maps in functional imaging: a general linear approach. *Human Brain Mapping*.

[10] Bagarinao E, Matsuo K, Nakai T, Sato S (2003). Estimation of general linear model coefficients for real-time application. *NeuroImage*.

[11] Roche A, Lahaye PJ, Poline JB Incremental activation detection in fMRI series using Kalman filtering.

[12] Averbeck BB, Lee D (2006). Effects of noise correlations on information encoding and decoding. *Journal of Neurophysiology*.

[13] Mattingely J, Boyd S (2010). Real-time convex optimization in signal processing. *IEEE Signal Processing Magazine*.

